# Stress circuitry: mechanisms behind nervous and immune system communication that influence behavior

**DOI:** 10.3389/fpsyt.2023.1240783

**Published:** 2023-08-29

**Authors:** Rose L. Tong, Ubaidah N. Kahn, Laura A. Grafe, Frederick L. Hitti, Nathan T. Fried, Brian F. Corbett

**Affiliations:** ^1^Corbett Laboratory, Department of Biology, Rutgers University, Camden, NJ, United States; ^2^Fried Laboratory, Department of Biology, Rutgers University, Camden, NJ, United States; ^3^Grafe Laboratory, Department of Psychology, Bryn Mawr College, Bryn Mawr, PA, United States; ^4^Hitti Laboratory, Department of Neurological Surgery and Psychiatry, University of Texas Southwestern Medical Center, Dallas, TX, United States

**Keywords:** stress, immunity, inflammation, mood, behavior, norepinephrine

## Abstract

Inflammatory processes are increased by stress and contribute to the pathology of mood disorders. Stress is thought to primarily induce inflammation through peripheral and central noradrenergic neurotransmission. In healthy individuals, these pro-inflammatory effects are countered by glucocorticoid signaling, which is also activated by stress. In chronically stressed individuals, the anti-inflammatory effects of glucocorticoids are impaired, allowing pro-inflammatory effects to go unchecked. Mechanisms underlying this glucocorticoid resistance are well understood, but the precise circuits and molecular mechanisms by which stress increases inflammation are not as well known. In this narrative review, we summarize the mechanisms by which chronic stress increases inflammation and contributes to the onset and development of stress-related mood disorders. We focus on the neural substrates and molecular mechanisms, especially those regulated by noradrenergic signaling, that increase inflammatory processes in stressed individuals. We also discuss key knowledge gaps in our understanding of the communication between nervous and immune systems during stress and considerations for future therapeutic strategies. Here we highlight the mechanisms by which noradrenergic signaling contributes to inflammatory processes during stress and how this inflammation can contribute to the pathology of stress-related mood disorders. Understanding the mechanisms underlying crosstalk between the nervous and immune systems may lead to novel therapeutic strategies for mood disorders and/or provide important considerations for treating immune-related diseases in individuals suffering from stress-related disorders.

## Introduction

Stress is well known to initiate and exacerbate mood disorders like anxiety, depression, and posttraumatic stress disorder (PTSD). These disorders are serious public health concerns as the lifetime prevalence rates for major depressive disorder (MDD), anxiety disorders, and PTSD are estimated to be estimated to be 10%–20%, 25%–31%, and 6%–8%, respectively (1–5). These disorders are closely related as the lifetime comorbidity of MDD and anxiety is over 60% and 50%–70% of individuals diagnosed with PTSD meet the criteria for anxiety and/or depression (3). All stress-related mood disorders tend to be approximately twice as prevalent in women compared to men, making sex a key factor underlying vulnerability to the adverse effects of stress (6).

A number of factors contribute to the onset and development of stress-related mood disorders. Socioeconomic factors (7), a history of stressors (8, 9), and employing passive coping strategies or substance abuse (10) represent extrinsic factors that promote stress vulnerability. Biological factors also contribute to stress vulnerability. The Val66Met polymorphism of the gene encoding brain derived neurotrophic factor (BDNF) impairs BDNF trafficking and its activity-dependent secretion (11). BDNF is important for promoting adult neurogenesis (12) and dendrite arborization (13) in the hippocampus. Therefore, this polymorphism results in reduced volume of the hippocampus and is associated with an increased risk for depression in individuals exposed to early life stress (14). Twin studies have also revealed that reduced hippocampal volume is also a risk factor for PTSD (15). Hippocampal neurogenesis and dendrite growth are considered two of the most important mechanisms underlying the efficacy of selective serotonin reuptake inhibitors (SSRIs), which represent the most commonly prescribed class of drugs prescribed for depression. SSRIs inhibit serotonin transporters, thereby reducing serotonin reuptake from the synaptic cleft and prolonging serotonergic neurotransmission. Polymorphisms in the promoter region of the gene encoding the serotonin transporter are associated with an increased risk for depression following multiple stressful life events (8). Interestingly, this effect is strengthened in individuals with the autoimmune disease multiple sclerosis (16). Despite the efficacy of SSRIs in treating depression, they may only be more efficacious than placebo in individuals suffering from severe depression (17). Therefore, impaired serotonergic neurotransmission is not the only factor contributing to stress-related mood disorders. Other biological factors must be considered to treat stress-related disorders, which develop from heterogeneous pathologies.

Depression, anxiety, and PTSD are all linked to increased inflammatory markers (18–21). Inflammation leads to sickness behavior, which is characterized by lethargy, reduced social interaction, anhedonia, and sleep impairments, which are also symptoms of depression (22, 23). Within the past decade we have learned that inflammatory processes contribute to depression in certain individuals. Non-steroidal anti-inflammatory drugs (NSAIDs) and pharmacological inhibitors of specific cytokines like tumor necrosis factor α (TNFα) reduce depression scores (24). The TNFα inhibitor infliximab reduces depression scores, but only in a subpopulation of individuals with higher levels of the inflammatory marker C-reactive protein (25). Therefore, inflammatory processes contribute to the pathology of stress-related disorders in certain individuals. Most studies linking inflammation with stress-related disorders focus on inflammatory markers in the blood. Peripheral cytokines can access the brain through multiple mechanisms including, but not limited to, specific cytokine transporters and a leaky blood brain barrier (BBB) caused by stress and inflammatory molecules in the blood (26). Indeed, inflammatory cytokine levels are increased in the brains of individuals suffering from depression (27) and animal models of stress (28, 29). Inflammatory molecules can affect neuronal activity by reducing serotonin levels and altering neurotransmitter function (30, 31). Together, these findings demonstrate that stress-related mood disorders are associated with increased inflammatory processes, which contribute to their pathology.

The immune system is tightly regulated by the stress response. The stress response activates two physiological systems: (1) noradrenergic signaling via the sympathetic nervous system and locus coeruleus (32) and (2) glucocorticoid signaling via the hypothalamic–pituitary–adrenal (HPA) axis (33). Noradrenergic neurotransmission is an immediate response to stress. Stress activates the sympathetic nervous system and the locus coeruleus to increase noradrenergic signaling in the periphery and brain, respectively. The HPA axis represents the neuroendocrine stress response, resulting in increased levels of circulating glucocorticoids. This is a relatively delayed response compared to noradrenergic neurotransmission. Stress-induced increases in circulating glucocorticoid levels are observed on the order of minutes (34). These pathways regulate the immune system with opposing effects. Noradrenergic neurotransmission is generally pro-inflammatory (35–37) whereas glucocorticoid signaling is anti-inflammatory (38, 39). Acute stress can cause temporary increases in inflammation (40), which return to baseline levels relatively quickly due to the anti-inflammatory effects of glucocorticoids. However, chronic stress causes glucocorticoid resistance, in which the anti-inflammatory effects of glucocorticoids are impaired (41). This tips the balance toward systemically increased inflammation. This inflammation can then create a positive-feedback loop that exacerbates these mood disorders and increases the impact of stress on the immune system in patients suffering from other conditions. While the mechanisms behind each of these systems are well-studied, there is a growing appreciation for how they interact to explain clinical phenomena and lead to the onset and development of mood disorders in vulnerable populations.

The main objectives of this narrative review are to: (1) summarize our current understanding of the mechanisms by which stress regulates inflammatory processes, (2) provide mechanisms by which inflammatory molecules can influence neuronal activity and mood, (3) describe how inflammatory pain might exacerbate inflammatory processes and contribute to negative mood, (4) highlight the role of glucocorticoid receptors in mitigating stress-induced inflammation, and (5) discuss knowledge gaps in our understanding of the neural circuits responsible for communication between immune and nervous systems. We discuss the physiological stress response, the effects of acute stress on inflammatory markers, and a key transcriptional mechanism underlying stress-induced inflammation. We then discuss the effects of inflammatory processes on neuronal activity and provide evidence that inflammation directly contributes to depression in certain individuals. We discuss how inflammation can be exacerbated by the neural substrates underlying nociception as inflammatory molecules sensitize pain-sensing neurons, which in turn release pro-inflammatory neuropeptides. We highlight the importance of glucocorticoids in mitigating inflammatory processes caused by stress and discuss how glucocorticoid signaling within the optimal range is necessary for resilience to the adverse effects of stress. We discuss key gaps in our understanding of the neural circuits and molecular mechanisms that contribute to stress-induced inflammation. We also discuss how this knowledge can potentially be used to develop novel therapeutic strategies, why this is currently challenging, and how these challenges might be overcome. Understanding the communication between the nervous and immune systems will allow basic scientists and clinicians to better understand this stress-immune axis and how it impacts biological processes, behavior, and patient outcomes.

### The physiological stress response

Stress can have profound effects on physiological, molecular, and neuronal mechanisms that lead to long-lasting changes in behavior. Physiological and psychological stressors activate multiple systems simultaneously, with one of the most immediate responses being increased noradrenergic signaling. In the periphery, norepinephrine is released from postganglionic neurons that regulate the sympathetic nervous system to increase heart rate and prime the organism for “fight or flight” (42). The primary source of norepinephrine in the brain is the locus coeruleus (6). Here, norepinephrine contributes to regulating a number of cognitive functions relevant to ensuring immediate survival including arousal, attention, and cognitive flexibility (43, 44). Sympathetic tone, assessed by increased heart rate variability, is elevated in individuals suffering from depression (45), anxiety (46), and post-traumatic stress disorder (PTSD) (47). Increased sympathetic tone is a key feature of the hyper-arousal symptom cluster in PTSD (47). Locus coeruleus activity correlates with stress and stress vulnerability as it predicts anxiety and depression scores (48). In rodents, the locus coeruleus is activated by a wide range of acute or chronic stressors (49, 50). Notably, locus coeruleus activity can be increased by corticotropin releasing factor (CRF) (51, 52), an initiator of the neuroendocrine branch of the stress response (33), which is activated by stress in parallel with noradrenergic signaling.

The hypothalamic–pituitary–adrenal (HPA) axis represents the neuroendocrine branch of the stress response. Here, a stressor increases the activation of parvocellular neurons in the paraventricular nucleus of the hypothalamus (PVN), which release CRF (53), thereby initiating HPA axis activation. PVN neurons project to blood vessels in the median eminence, which deliver CRF to the anterior pituitary. CRF binding to its receptors on corticotropic cells in the anterior pituitary triggers the calcium-dependent cleavage of adrenocorticotropic hormone (ACTH) from pro-opiomelanocortin (54). ACTH is released into blood circulation where it binds to MC2 and 3 receptors in the zona fasciculata of the adrenal gland (55). These G protein coupled receptors increase intracellular cyclic adenosine monophosphate (cAMP) concentrations, leading to the activation of protein kinase A and increased expression of enzymes responsible for converting cholesterol to glucocorticoids like corticosterone (rodents) and cortisol (humans) (56). Corticosterone binds to mineralocorticoid (MRs) and glucocorticoid receptors (GRs) (57, 58), which are expressed in a wide range of cell types including neurons and immune cells in the brain and periphery (56). Once bound and activated, GRs bind to glucocorticoid response elements throughout the genome and recruit co-activators or co-repressors to modulate gene expression (39). MRs have a higher affinity for corticosterone than glucocorticoid receptors do (56, 59). Because of this, mineralocorticoid receptors are saturated at baseline glucocorticoid levels, so the effects of stress-induced glucocorticoids are primarily mediated by GRs (57). GRs regulate a number of functions including, but not limited to, altering the structure and function of neurons (60–62), inducing metabolic changes (63), and reducing inflammation (38).

Thus, in humans and rodents, chronic stress activates both noradrenergic signaling and the HPA axis. Since a major result of noradrenergic signaling is an increase in inflammatory markers, an important role for GR signaling is the compensatory reduction of inflammatory processes (39). These systems are generally balanced in response to mild or acute stress. In response to chronic stress, the anti-inflammatory effects of glucocorticoids can become less effective. This, along with excess noradrenergic signaling, is believed to contribute to the increased inflammatory cytokine levels observed in stress-related mood disorders.

### Acute psychological stress increases inflammatory markers

Acute stressful experiences result in increased inflammatory marker expression and other neurophysiological changes. The Trier Social Stress Test (TSST) is commonly used as an acute psychological stressor in humans (64). In this public speaking task, participants are required to provide an interview-style presentation, followed by a surprise mental arithmetic test in front of a panel that does not provide feedback or encouragement. Indeed, the TSST increases HPA axis activation (65) and noradrenergic signaling as the sympathetic nervous system is activated (66). The TSST is associated with increased transcriptional activity of nuclear factor kappa B (NFkB) (35, 67). NFkB is a master transcriptional regulator of inflammatory processes. Following its activation via translocation to the nucleus, NFkB increases the expression of a wide range of inflammatory transcripts while suppressing anti-inflammatory gene expression through multiple mechanisms, including inhibiting GR-mediated transcription (68). Increased inflammation during the TSST is likely due to noradrenergic signaling regulated by the sympathetic nervous system as norepinephrine increases NFkB-mediated transcription (35). NFkB activity inversely correlates with cortisol levels during the TSST (67), which may be attributed to the direct and indirect mechanisms by which GRs inhibit NFkB (39, 69). GRs suppress the activity of NFkB (68–70) via direct binding that prevents NFkB interactions with DNA, by recruiting co-repressors to pro-inflammatory transcriptional sites, through competition for co-activators of NFkB, and by increasing the expression of proteins that inhibit NFkB function (41, 71). NFkB, in turn, inhibits GRs by similar mechanisms (69).

A meta-analysis of 34 studies measuring circulating inflammatory markers indicate that in individuals exposed to acute stress under laboratory conditions, levels of interleukin (IL)-1β, IL-6, and tumor necrosis factor α (TNFα) are increased (72). Acute stressors in this meta-analysis included recall tasks, mock interviews, and Stroop tasks along with being put under social evaluative stressors like the TSST. Peripheral blood samples taken before and after stressors revealed an increase in inflammatory markers (72). Acute stress also increases inflammatory processes in other peripheral fluids such as gingival crevicular fluid, in which IL-1 β is increased (73).

Single and multiple stressors also increase inflammatory markers in rodents. In established rodent stress models, such as social defeat stress, inflammatory processes are activated in the brain and periphery. A single social defeat session in the resident-intruder paradigm increases IL-6 mRNA expression in the medial prefrontal cortex (mPFC), while increases in IL-1β and TNFα are only observed in the mPFC after at least 7 days of stress (28). The mPFC is an important stress-related brain region as it provides top-down negative control of the HPA axis (74) and the amygdala (75), a brain region important for fear and anxiety. Therefore, the mPFC is important for suppressing neuroendocrine and behavioral responses to stress. The mPFC is also important for cognitive flexibility (76) and resilience to stress (77). We demonstrated that IBA1+ cell densities and TNFα are increased in the mPFC of vulnerable rats, which display increased anxiety- and depression-like behavior following 7 days of social defeat in the resident-intruder paradigm. Levels of these inflammatory markers in the mPFC of resilient rats, which behave similarly to non-defeated controls, are lower and similar to those of control rats (29). Indeed, chronic administration of infliximab, a TNFα antagonist, reduces anxiety- and depression-like behavior following chronic stress in rodents (78).

Mood disorders associated with chronic stress are also associated with increases in inflammatory markers in the blood (18, 20, 27, 79, 80) and brain (27). Veterans with PTSD from combat exposure have higher pro-inflammatory cytokine levels than veterans that did not develop PTSD following combat (80). The inflammatory markers TNF-α and IL-6 are also higher in the blood of individuals with major depression (79), and generalized anxiety disorder (GAD) (19, 20). In suicide victims, mRNA and protein levels of the pro-inflammatory cytokines IL-6, IL-1β, TNF-α and lymphotoxin A were increased in the prefrontal cortex (PFC) (27). Individuals who have had autoimmune diseases that can dysregulate the immune response are 45% more likely to develop depression (81). Therefore, chronic stress disorders are associated with increased inflammation and auto-immune disorders can lead to stress-related mood disorders in humans. These increases in inflammatory cytokines are not only symptomatic of immune dysfunction and systemic inflammation, but contribute to symptoms of depression.

### Inflammation directly affects mood disorders

Inflammatory cytokines are not only increased by stress but contribute to the pathology of stress-related mood disorders. A broad range of social (8), anatomical (15), and molecular (82, 83) factors contribute to the onset and development of stress-related mood disorders. It is well-established that inflammatory markers are increased in the blood (79) and brain (27) of individuals afflicted with stress-related mood disorders. Moreover, blocking inflammation can reduce phenotypes associated with stress-related mood disorders (24, 25), whereas increasing inflammatory processes can contribute to the onset and development of stress-related mood disorders (84, 85).

For example, a meta-analysis of 14 trials with 6,262 participants revealed that pharmacological cytokine inhibitors or non-steroidal anti-inflammatory drugs (NSAIDS) can reduce depression scores in adults with depressive symptoms compared to placebo controls (24). A subsequent meta-analysis showed similar anti-depressant effects of NSAIDs in trials that included both men and women (86). In a randomized trial of participants with treatment-resistant depression, the TNFα inhibitor infliximab reduces depression scores, but only in the subpopulation of patients displaying elevation of the inflammatory marker C-reactive protein (25). Similar findings have been observed in preclinical studies as weekly intraperitoneal injections of infliximab reduce depression- and anxiety-like behavior in rats following an 8-week chronic mild stress paradigm (78). Therefore, anti-inflammatory treatments reduce symptoms of depression in certain human populations and rodents.

Conversely, treatments using exogenous cytokines increase symptoms of depression. Interferon-α (IFNα) exacerbates symptoms of depression when used to treat hepatitis C (84). IFNα also increases symptoms of depression when used to treat melanoma (87, 88). SSRIs can reduce symptoms of depression in hepatitis C and cancer patients receiving IFNα treatments (89, 90), suggesting that IFNα might increase depression severity by reducing serotonergic neurotransmission. In rodents, induction of inflammatory processes using intraperitoneal lipopolysaccharide (LPS) administration increases depressive-like behavior in mice as characterized by immobility in the forced swim test and tail suspension test (91). Therefore, exogenous cytokines are sufficient to induce symptoms of depression in humans and mice.

Studies in the absence of any treatments also support a relationship between stress-related mood disorders and immune dysfunction as depression and autoimmune disorders share comorbidity, common symptoms, and inflammatory processes (92). Diseases characterized by increased peripheral inflammation, like rheumatoid arthritis (93) and sepsis (81) have been linked to an increased risk for depression. Together, these findings indicate that inflammatory processes contribute to symptoms of depression in certain individuals and are sufficient to induce or exacerbate depressive symptoms.

### Stress promotes inflammatory processes via NFkB

Stress-induced noradrenergic signaling can promote inflammatory processes (35, 37, 94). Noradrenergic signaling can be pharmacologically inhibited using the α1 adrenergic receptor antagonist prazosin or the β adrenergic receptor antagonist propranolol. The α1 adrenergic receptors are G_q/11_ protein coupled receptors (GPCRs) that activate phospholipase C β, which increases concentrations of diacylglycerol and inositol trisphosphate, thus activating protein kinase C signaling and increasing intracellular calcium concentrations. The β adrenergic receptors are G_s_PCRs that activate adenylyl cyclase, converting adenosine triphosphate to 3′,5′-cyclic adenosine monophosphate (cAMP), which activates protein kinase A signaling (95). Norepinephrine, but not epinephrine, increases peripheral inflammation as it induces NFkB-mediated transcription in mononuclear cells (35). Indeed, antagonism of α1 adrenergic receptors using prazosin can reduce inflammatory markers in the blood of PTSD patients (96). In mice, the β adrenergic receptor antagonist propranolol mitigates social defeat-induced microglial reactivity in the prefrontal cortex, the hippocampus, and the amygdala (37) and foot shock-induced increases in IL-1β in the hypothalamus (36). Each of these brain regions receive noradrenergic input from the locus coeruleus (97). Propranolol also prevents stress-induced IL-6 elevation in healthy young adult humans (40). PTSD symptom severity is associated with both increased sympathetic tone and inflammation (98), which is consistent with systemic inflammation being driven by noradrenergic neurotransmission in humans. Together, these findings indicate that noradrenergic neurotransmission, which is increased by stress in the brain and periphery, increases peripheral and central inflammatory processes.

The context of psychological stress might be an important factor contributing to the pro-inflammatory effects of norepinephrine. In response to certain inflammatory stimuli, such as exposure to lipopolysaccharides, norepinephrine can provide anti-inflammatory effects (99). Norepinephrine can also inhibit microglia reactivity, at least in certain mouse models of Parkinson’s disease (100). Generally, G_q_-coupled α1 and G_i_-coupled α2 adrenergic receptors are thought to mediate pro-inflammatory effects (101). G_s_-coupled β adrenergic receptors may mediate anti-inflammatory effects by inhibiting nuclear translocation of NFkB through activation of protein kinase A induced by cAMP (102, 103). However, β adrenergic receptors have been proposed to adopt non-canonical mitogen-activated protein kinase (MAPK) pathways that can increase inflammatory processes (104). These findings underscore the complex nature by which norepinephrine affects inflammation. Additional work is needed to better understand the contexts, molecular states, and immune cell types in which noradrenaline primarily exerts anti- vs. pro-inflammatory effects.

Norepinephrine is the most extensively studied signaling molecule to be implicated in stress-induced inflammation, although other singling molecules increased by stress also contribute to inflammatory processes. Stress-induced CRF release has been studied most extensively in PVN neurons. However, stress can also increase CRF release in the prefrontal cortex (105), amygdala (106), and periphery (107, 108). In the periphery, CRF increases inflammation through direct interactions with macrophages in cell lines derived from mouse myeloma cells (107). CRF potentiates the pro-inflammatory effects of LPS as TNF-α, IL-1β, and IL-6 concentrations are higher in the presence of CRF and LPS compared to LPS alone (107). The neuropeptide substance P is increased by certain stressful stimuli, like loud sounds (109) and cold water (110). Substance P stimulates cytokine secretion from peripheral immune cells during stress (110) and in the absence of stress (111). Substance P also exerts pro-inflammatory effects in the central nervous system as it increases nuclear translocation of NFkB in cultured microglia (112). Pro-inflammatory effects of substance P in the periphery and central nervous system have been previously reviewed (113). Therefore, like noradrenergic signaling, stress-induced CRF and Substance P signaling can promote inflammatory processes in the periphery and brain.

### Inflammation influences neuronal activity via peripheral and central mechanisms

Stress increases the expression and release of inflammatory molecules, which modulate neuronal activity to affect mood and behavior. Inflammatory processes can induce sickness behavior, characterized by anhedonia, reduced motivation, lethargy, reduced social interaction, and altered sleep architecture. These behaviors share similarity with symptoms of MDD (22). In addition to central inflammatory processes induced by locus coeruleus-derived noradrenergic signaling, peripheral inflammation resulting from sympathetic nervous system activity can also affect the brain. While peripherally and centrally derived cytokines likely affect neuronal activity similarly, peripheral cytokines must first cross the blood–brain barrier (BBB). Peripheral cytokines can access the brain through circumventricular organs, specific cytokine transporters, recruitment of peripheral immune cells by reactive microglia, a leaky BBB caused by stress and/or prior inflammation, and other mechanisms. These mechanisms have been reviewed extensively by others (22, 23, 26, 114).

Cytokines have direct effects on neuronal activity. In response to inflammation, interferons and interleukins are released and levels of indoleamine 2,3 deoxygenase (IDO) are increased. IDO is responsible for catabolizing tryptophan into kynurenine, which further catabolizes into metabolites like quinolinic acid in microglia and kynurenic acid in astrocytes (30, 31, 115). Because tryptophan is a key precursor for serotonin, this reduces the amount of tryptophan available for serotonin synthesis, thus reducing levels of serotonin in the brain (31). Quinolinic acid can have excitotoxic effects via activation of N-methyl-D-aspartate (NMDA) receptors whereas kynurenic acid can antagonize NDMA receptors and α7 nicotinic acetyl choline receptors (30). Inflammatory cytokines can also reduce serotonergic neurotransmission by promoting serotonin reuptake as IL-1 β increases serotonin transporter (SERT) activity (116). These mechanisms, especially those reducing serotonergic neurotransmission, can promote depression- and anxiety-like behavior as the role of serotonin in regulating mood is well-documented (114).

Increased serotonergic neurotransmission can facilitate neurogenesis in the subgranular zone of the dentate gyrus (117), which is important for the antidepressant effects of selective serotonin reuptake inhibitors (SRRIs) (118). Indeed, stress-induced IL-1 β release contributes to depression-like behavior and reduced neurogenesis as pharmaceutical inhibition or genetic knockout of the IL1 receptor prevents anhedonia-like behavior and anti-neurogenic effects in a mouse model of chronic stress (119). The inflammatory cytokine interferon-α can also reduce dopamine levels by reducing levels of one its precursors, tyrosine, and metabolizing dopamine directly (120). Several other neurotransmitters can also be impacted via similar mechanisms to control mood and brain function (114).

### Inflammatory pain increases stress and impacts mood disorders

The neural substrates underlying pain represent an important, and often overlooked, system that can cause a positive feedback loop that increases inflammation during stress. A heightened sense of pain represents one of the most prominent symptoms caused by inflammation. Pain is an unpleasant sensory and emotional experience caused by actual or potential tissue damage. While the sensation originates with activation of nociceptors throughout the periphery, the signals are sent to the brain via the spinothalamic tract and further shaped by supraspinal brain regions that encode it with a negative emotional valence (121). Interestingly, many of the stress-related brain areas such as the locus coeruleus, PVN, hippocampus, amygdala, and mPFC are also involved in pain processing and play a role in the transition to chronic pain (122–125). The locus coeruleus, which provides analgesic effects during acute pain, has been proposed to contribute to allodynia and hyperalgesia in the context of chronic pain (125). Despite projections of nociceptive pathways to the PVN, chronic pain models do not elicit changes in HPA axis activation. However, chronic pain causes changes in limbic regions associated with stress and mood (126). Indeed, reduced hippocampal volume is a biomarker for both chronic pain (127, 128) and depression (129). It is unclear whether reduced hippocampal volume associated with chronic pain is attributed to nociceptive spinothalamic pathway projections or secondary effects caused by increases in inflammatory cytokines, which are known to reduce neurogenesis and dendrite growth in the hippocampus (130). Regardless of the mechanism(s) responsible, reduced hippocampal volume caused by pain likely contributes to aspects of depression due to the importance of hippocampal volume in promoting stress resilience (15). Conversely, chronic pain increases the activity of the amygdala, which regulates fear and anxiety-like behavior. This is at least partially due to dysfunction of GABAergic neurons within the amygdala, allowing for enhanced plasticity and facilitated excitatory inputs from the parabrachial nucleus, which transmits nociceptive information from the periphery. Heightened activity within the amygdala also contributes to inhibition of the mPFC (131). The mPFC is involved in a wide range of functions including attention (132), cognitive flexibility (76), stress resilience (29, 77) and negative regulation of the HPA axis (74). Therefore, pain modulates the activity of multiple stress-related brain regions that might contribute to depression- and anxiety-like behavior.

While nociceptors can be activated by noxious chemical, thermal, and mechanical stimuli at baseline, local inflammation from injury or infection can sensitize them. Mast cells, macrophages, and keratinocytes release inflammatory mediators such as TNF-α, IL-1β, and IL-6 that increase infiltration of immune cells that further promote inflammation (133). These mediators increase hyperactivity of nociceptors by activating intracellular signaling pathways that increase sensitivity and expression of sodium channels and nociceptor-specific ion channels such as the Transient Receptor Potential Vanilloid Type 1 (TRPV1) receptor and Transient Receptor Potential Ankyrin Type 1 (TRPA1) receptor (134). Indeed, initiating these inflammatory processes with the injection of formalin, complete Freund’s adjuvant (CFA), carrageenan, or an inflammatory soup (i.e., an experimental mixture of serotonin, bradykinin, prostaglandin E2, and histamine) results in acute and long-term pain-related behavior in rodents (135, 136). Interestingly, this inflammatory sensitization at the periphery is bidirectional as substance P and calcitonin gene-related peptide (CGRP), which are important for neurotransmission in nociceptive neurons, further increase inflammation (137). Indeed, substance P increases inflammatory markers in the periphery (110, 111) as well as cultured brain cells (112, 113). Even in the absence of tissue injury, aberrant neuronal signaling can induce inflammation and swelling of tissue (138). Therefore, cross-talk between the immune system and nociceptive neurons is reciprocal, which might lead to positive feedback cycles that exacerbate inflammatory processes in chronically stressed individuals.

Repeated activation of these nociceptors under chronic pain conditions can also cause microglia reactivity, leading to central sensitization of nociceptive pathways. Here, microglia reactivity is thought to occur due to the release of the chemokine CCL2 from the chronically active nociceptor (134). Once reactivate, microglia enter a state of microgliosis and release pro-inflammatory mediators such as IL-1β and TNF-α. These sensitize the pre-synaptic nociceptor and post-synaptic second-order neuron while also activating astrocytes that further release pro-inflammatory cytokines. Given the involvement of microglia and astrocytes in the maintenance of the BBB, chronic inflammatory pain leads to an increase in BBB permeability (139), allowing peripheral cytokines to access the brain parenchyma more easily. Interestingly, these neuroinflammatory processes do not appear to occur under acute conditions and thus may be important for the maintenance of pain under chronic conditions (140).

Understandably, treating inflammatory pain with non-selective COX inhibitors and other non-steroidal anti-inflammatory drugs (NSAIDs) is considered first-line therapy for clinicians (141). Other treatments such as infliximab, glucocorticoids, and minocycline are also effective for pain, presumably through modulating peripheral and central inflammatory processes (142–145). Surprisingly, however, several other therapeutics that target stress-related systems also show efficacy in treating inflammatory pain. Topical prazosin reduced allodynia and hyperalgesia in patients with complex regional pain syndrome (146). Propranolol reduced pain in a larger percentage of patients with temporomandibular disorder (TMD) in a randomized placebo-controlled clinical trial and reduced temporomandibular joint pain in a female rats via anti-inflammatory mechanisms (147, 148). Antidepressants including monoamine oxidase inhibitors, tricyclic antidepressants, monoamine reuptake inhibitors, and glutamatergic antidepressants have all shown efficacy in treating multiple forms of chronic pain (149). Indeed, it is difficult to separate the psychological components of stress, depression, and pain when seeking to understand the mechanisms behind therapeutic strategies that have efficacy in all three disorders. Given that these all these antidepressants affect inflammatory mechanisms, however, it suggests stress-induced inflammation could be a key contributor to their shared efficacy (150).

### Glucocorticoid resistance & treatment for stress-related disorders

Impairments in the anti-inflammatory mechanisms regulated by GRs can lead to systemic inflammation in chronically stressed individuals. Negative feedback of the HPA axis prevents continuous activity of itself during periods of prolonged stress. Here, negative feedback is mediated by glucocorticoids, which bind to glucocorticoid receptors in multiple tissues including the anterior pituitary (151), PVN (152), and PFC (74). Negative feedback of the HPA axis is overactive in individuals with PTSD, resulting in lower levels of circulating glucocorticoids in the absence of stress (153). In some depressed individuals, glucocorticoid resistance is observed as dexamethasone-induced anti-inflammatory effects are less efficacious (154, 155). Glucocorticoid resistance is widely believed to be an important factor contributing to systemic inflammation observed in stress-related psychiatric disorders. This impaired ability of glucocorticoid receptors to exert anti-inflammatory processes can leave pro-inflammatory mechanisms, such as increased noradrenergic signaling, to go unchecked. Notably, cytokines can also contribute to glucocorticoid resistance (156), causing a positive feedback loop that exacerbates inflammatory processes during chronic stress.

A number of factors can cause glucocorticoid resistance. Glucocorticoid signaling can be impaired by DNA methylation of the gene encoding GRs, thereby reducing GR expression (157). Additionally, previous exposure to stress or glucocorticoids reduces the affinity of GRs for glucocorticoids during subsequent exposure to glucocorticoids (158). Glucocorticoid resistance can also be caused by impaired nuclear translocation of GRs. GRs that are not bound to cortisol or corticosterone are retained in the cytoplasm as part of a complex that includes multiple proteins including, but not limited to, heat shock protein 70 (HSP70), p23, and FK506-binding protein 51 (FKBP5) (159). Upon binding of GRs to cortisol or corticosterone, FKBP5 is replaced with FKBP4, which interacts with the motor protein dynein. This allows for the translocation of the GR-bound complex toward the nucleus along microtubules (160, 161). Once in the nucleus, GRs regulate the expression of thousands of genes, including FKBP5. Induction of FKBP5 by GRs allows for a short negative feedback loop whereby GR-mediated transcription impairs GR nuclear translocation. Stress increases FKBP5 expression in the rodent prefrontal cortex and hippocampus, which impairs nuclear translocation of GRs and the expression of genes regulated by GRs (162). Thus, FKBP5 is considered to be an important factor contributing to glucocorticoid resistance (163). Interestingly, these molecular mechanisms involving FKBP5-mediated glucocorticoid resistance are returned to normal expression and function following SSRI treatment (162). Therefore, this mechanism might contribute to the antidepressant effects of SSRIs.

**Figure 1:**
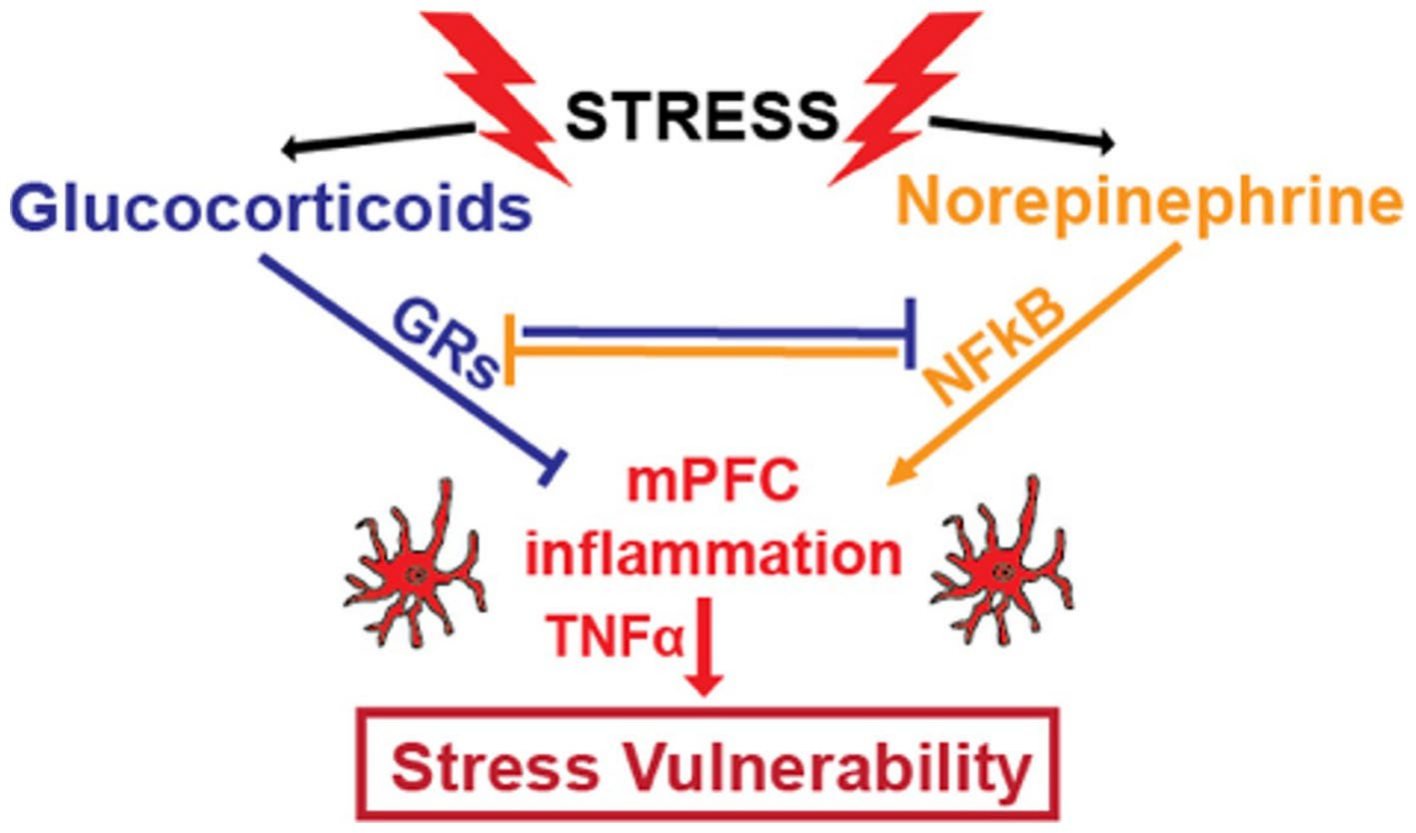
Stress increases glucocorticoid concentrations by activating the HPA axis and increases noradrenergic signaling by increasing activity of the sympathetic nervous system and locus coeruleus. Glucocorticoids and norepinephrine have counter-regulatory effects on inflammatory processes. Glucocorticoid receptors (GRs), activated by binding to glucocorticoids, and nuclear factor kappa B (NFkB), whose transcriptional activity is increased by noradrenergic signaling, functionally inhibit one another through multiple mechanisms. By directly and indirectly inhibiting NFkB, a master transcriptional regulator of inflammatory genes, GRs provide anti-inflammatory effects. In healthy individuals, these two stress-induced systems are generally balanced. However, chronic stress can disrupt this balance through chronic increases in noradrenergic neurotransmission and/ or by decreasing the anti-inflammatory efficacy of glucocorticoids through glucocorticoid resistance. This results in increased levels of pro-inflammatory cytokines like TNFα, which contributes to symptoms of depression and anxiety.

Glucocorticoid resistance can also be caused by NFkB, which impairs the ability of GRs to bind DNA and regulate gene expression (38, 164). NFkB can suppress GR function via direct binding that prevents GR binding to DNA, by outcompeting GRs for co-activators, and by recruiting co-repressors to GR-binding sites (69, 70). GRs inhibit NFkB via similar mechanisms (41, 71, 165, 166). Therefore, pro- and anti-inflammatory mechanisms that are activated by stress are normally in balance. In chronically stressed individuals, this reciprocal inhibition can be disturbed. This can lead to glucocorticoid resistance and positive feedback cycles that increase inflammatory processes (Figure 1).

Aberrant glucocorticoid signaling is a hallmark of stress-related psychiatric disorders. MDD patients display increased salivary cortisol in the first hour of wakefulness, but salivary cortisol concentrations are similar to controls within 8 h of wakefulness (167). Because the glucocorticoid resistance experienced by individuals with MDD is likely continuous, overall glucocorticoid signaling might be reduced in depressed individuals, despite increased cortisol early in the day. In a randomized trial, dexamethasone was shown to improve Hamilton Depression Rating Scale scores in depressed patients compared to placebo over the course of 4 days (168), suggesting that impaired GR activation might contribute to symptoms of depression in some MDD patients. PTSD is characterized by reduced baseline cortisol levels, (169) which can be attributed to hypersensitivity to glucocorticoid-mediated negative feedback of the HPA axis (153). Low-dose dexamethasone treatment can reduce inflammatory markers in PTSD patients and reduce the severity of certain PTSD symptoms (19).

Recently, we found that GR expression is increased in the mPFC of rats resilient to the adverse behavioral effects of social defeat. We found that a specific target of GRs, sphingosine-1-phosphate receptor 3 (S1PR3), prevented anxiety- and depression-like behavior by mitigating stress-induced inflammation. S1PR3 mRNA was also reduced in the blood of veterans with PTSD (29), which is consistent with increased inflammatory markers in PTSD. Conditional knockout of GRs in the forebrain causes depression-like behavior in rodents (170). Of course, GRs reduce inflammatory processes in the periphery and brain through regulating many transcriptional targets. Inflammatory cytokines can affect neuronal activity, and likely mental health, by altering neurotransmission. Some of the adverse effects on mood caused by inflammatory cytokines may also involve pain, which has both physiological and psychological components. While glucocorticoids reduce pain caused by inflammation (145) and can improve measures of mental health at certain doses in some individuals (19, 168), whether glucocorticoids improve mental health by alleviating pain has not been directly investigated. Impaired GR function and circulating glucocorticoid levels outside the optimal range can contribute to symptoms of depression and PTSD. Of course, chronic increases in circulating glucocorticoid concentrations can contribute to a wide range of adverse biological and psychological effects, but glucocorticoid signaling also has positive effects on mental health in certain contexts. This includes, but is not limited to, mitigating stress-induced inflammatory processes prior to the development of glucocorticoid resistance.

### Dissecting stress circuits as a means of addressing knowledge gaps

Decades of research has identified many key molecular mechanisms underlying the interplay among stress, inflammation, pain, and behavior. The nervous system is central to this interplay, but we have yet to identify many of the circuits responsible for this interplay. For example, we know that noradrenergic neurotransmission contributes to inflammatory processes in immune cells of the blood and brain using pharmacology. These noradrenergic neurons are likely postganglionic sympathetic neurons and locus coeruleus neurons. However, it is possible that certain subregions of these noradrenergic centers are more important for stress-induced inflammatory processes than others. For example, thoracic, but not lumbar, sympathetic neurons might underlie stress-induced inflammation. We know that norepinephrine induces microglia reactivity and cytokine production and assume this norepinephrine is derived from the locus coeruleus, but this has yet to be directly demonstrated. We also do not know if the pro-inflammatory effects of norepinephrine in microglia are due to NFkB-mediated transcription as they are in peripheral immune cells.

The activity of noradrenergic neurons and specific neural circuits can be manipulated using chemogenetic or optogenetic methods. In particular, viral vectors expressing hyperpolarizing hM4D and depolarizing hM3D Designer Receptors Exclusively Activated by Designer Drugs (DREADDs) under the control of the PRSx8 promoter have been developed (171). These constructs allow for decreasing or increasing the activity of noradrenergic neurons within heterogenous neuronal populations. For the manipulation of neural circuits, Cre-dependent DREADDs can be used in combination with CAV2-Cre, which retrogradely expresses Cre recombinase. Here, Cre-dependent DREADDs are injected into a particular brain region and CAV2-Cre is injected in a target region of choice (172, 173). This allows DREADDs to be expressed in a subpopulation of neurons that project to a specific brain region. This technology is also an excellent tool for specifically targeting pain-related circuits, especially when used in combination with nociceptive neuron-specific promoters, such as the substance P promoter. Of course, optogenetic approaches can also be used, although DREADDs are typically better suited for stress paradigms as they avoid the use of cumbersome and sensitive lasers. When using Cre-dependent DREADDs, it is important to consider collateral projections, as these will also be inhibited. For example, using this system to chemogenetically inhibit mPFC-projecting locus coeruleus neurons will inhibit the entire locus coeruleus neuron, which may also innervate other brain regions via collateral projections. This can be avoided using optogenetic approaches, although this might not be conducive to stress paradigms involving physical contact between rodents.

## Discussion

Cross-talk between the nervous and immune systems during stress is complex and can have profound effects on behavior. Stress activates both the sympathetic nervous system and the HPA axis, which are generally pro- and anti-inflammatory, respectively (35, 60). Each of these systems are activated to satisfy the metabolic demands of dealing with a perceived physical or psychological threat. Tissue damage is a possible consequence of stress and may be caused by the stressor itself or secondary effects, like increased heart rate or blood pressure. This is particularly relevant to chronic stress, when these systems are continuously active, even in the absence of an immediate physical threat. As a nearly immediate response to stress, the sympathetic nervous system might stimulate the immune system to initiate healing of any potential tissue damage. The slower neuroendocrine stress response, which increases circulating glucocorticoids on the order of minutes (33), might provide anti-inflammatory effects as a means of (1) reallocating energy for other metabolic demands caused by the stressor and other functions regulated by glucocorticoids and/or (2) to mitigate excessive inflammation caused by norepinephrine. In healthy individuals, these major stress systems are balanced. However, chronic stress can disturb this balance via heightened activation of the sympathetic nervous system and/or through glucocorticoid resistance. This imbalance results in systemic increases in inflammatory markers, which contribute to the onset and development of stress-related psychiatric disorders (24) and underly comorbidities with physiological disorders like heart disease and irritable bowel syndrome (174, 175). In a subset of individuals diagnosed with Major Depression that also have high inflammatory markers, TNFα inhibition reduced depression scores (25). This work is promising and highlights the need for more personalized treatments for depression, as this disorder can be caused by many biological factors. RNA sequencing of inflammatory transcripts in specific cell types or targeted cytokine screens might provide more accurate biomarkers for subtypes of depression with distinct pathologies to allow for more personalized treatments.

NFkB and GRs represent two major transcription factors that regulate gene expression in response to activation of the sympathetic nervous system activation and the HPA axis, respectively. NFkB is a master transcriptional regulator of the inflammatory response whereas GRs are highly effective anti-inflammatory transcription factors, which is largely attributed to their inhibition of NFkB. GRs inhibit NFkB through direct interactions that prevent binding to DNA and indirect mechanisms, like increasing the transcription of proteins that prevent the nuclear translocation of NFkB. NFkB inhibits GRs through similar mechanisms (35, 38, 165). Glucocorticoid resistance impairs GR function, thereby disinhibiting NFkB and increasing the transcription of inflammatory genes (41). GRs and NFkB are both expressed in peripheral immune cells, neurons, and microglia. We know that acute psychosocial stress increases NFkB-mediated transcription in mononuclear immune cells via norepinephrine (35) and norepinephrine increases inflammatory processes in the brain (37). However, we do not know whether NFkB regulates stress-induced inflammation in other immune cells, including microglia, and/or is responsible for increased systemic inflammatory markers in chronically stressed individuals. We understand that noradrenergic neurotransmission increases inflammatory processes in the brain, but we lack a comprehensive understanding of the transcriptional mechanisms that are responsible. In sum, there is a knowledge gap in our understanding of stress-induced transcriptional mechanisms that increase inflammatory processes across cell types.

Activation of GRs can cause a number of deleterious effects, such as reducing hippocampal volume (61), which can contribute to depression (130). However, optimal function of GRs is also important in mitigating stress-induced inflammation, thereby preventing stress-induced behavioral changes. Glucocorticoid resistance impairs the anti-inflammatory effects of GRs, allowing the pro-inflammatory effects of NFkB to be disinhibited. Indeed, this contributes to inflammatory processes and symptoms of stress-related disorders as low-dose dexamethasone treatment reduces inflammatory markers and ameliorates certain symptoms of PTSD (20). We found that GRs are increased in the mPFC of rats that are resilient to the adverse effects of stress (29) and others have shown forebrain GRs reduce depression-like behavior (170). The mPFC is prone to stress-induced inflammatory processes in vulnerable rats and therefore GRs might promote resilience by mitigating these inflammatory processes. Indeed GR deletion in the forebrain of rodents induces depressive-like behavior (170). We lack a thorough understanding of why certain brain regions like the mPFC, hypothalamus, amygdala, locus coeruleus, and hippocampus are more prone to stress-induced inflammation than others (28, 37, 176–180). Possibilities include, but are not limited to, particularly strong noradrenergic inputs, high levels of noradrenergic receptors, and/or increased neuronal activity.

Stress-induced inflammation affects neuronal activity in the brain. Within the brain, locus coeruleus activation increases noradrenergic neurotransmission, which can increase inflammatory processes. Peripheral cytokines can also influence brain activity through direct and indirect mechanisms. Cytokines can directly access the brain via humoral routes through circumventricular organs, blood–brain barriers with impaired integrity caused by stress, and/or specific cytokine transporters. Peripheral cytokines can also influence brain activity by modulating vagus nerve activity (22, 23, 26, 114). In the brain, cytokines reduce the levels of neurotransmitters important for motivation and positive affect and modulate the activity of neurotransmitter receptors (22, 30). Inflammatory cytokines can also sensitize nociceptive neurons, which can influence mood (134). Certain nociceptive neurons, like those in the archispinothalamic pathway, project to limbic structures and are responsible for the negative emotions associated with pain. We lack a comprehensive understanding of how nociceptive circuits contribute to mood in response to stress-induced inflammation. Further, we do not understand the extent by which nociceptive neurons exacerbate stress-induced inflammation as certain neuropeptides released by nociceptive neurons, like substance P, can promote inflammatory processes.

Most of what we know about the effects of norepinephrine-induced inflammation comes from pharmacological studies. The locus coruleus is the primary source of norepinephrine in the brain (32). In the periphery, norepinephrine is primarily released from postganglionic sympathetic neurons (181). Because these sources of norepinephrine are so well defined it is generally assumed, and for good reason, that the effects of norepinephrine on inflammatory processes in the brain can be attributed to the locus coeruleus whereas effects in the periphery can be attributed to the sympathetic nervous system. However, the roles of these noradrenergic sources on inflammatory processes have not been fully demonstrated. Perhaps subregions of the postganglionic sympathetic column, such as regions that more densely innervate the vascular system, are more likely to increase inflammatory markers. This would be valuable as it would provide insight into the mechanisms contributing to stress-induced inflammation. This could be accomplished using chemogenetic or optogenetic methods to hyperpolarize or depolarize specific cell types or neural circuits. Understanding the specific neuronal populations contributing to stress-induced inflammation would provide a more comprehensive understanding for how inflammatory processes develop in response to stress. Understanding the role of norepinephrine on inflammatory processes is extremely important, especially because it has opposing roles on inflammation in differnet disease states. Norepinephrine is pro-inflammatory stressed rodents (37) and humans (40), but exerts anti-inflammatory effects in diseases like Parkinson’s disease (100) and sepsis (182). We need to better understand the timeframe, specific receptors, cell types, and intracellular signaling pathways by which norepinephrine regulates inflammation. This is challenging as it will require manipulation and/or screening of a wide range of genes within multiple cell types during different time points.

Novel therapeutic strategies for treating stress-related disorders could target mechanisms by which stress induces inflammatory processes or the specific inflmamamtory processes changed by stress. Targeting specific cytokines, especially within specific cell types or tissues, might lead to efficacious treatments with minimal side effects. Targeting TNFα systemically has demonstrated considerable promise (25). Future research focused on understanding the intracellular signaling mechanisms activated by adrenergic receptors might identify mechanisms that could be targeted pharmacologically. For example, it has been proposed that in chronic stress, non-canonical MAPK signaling pathways might be activated by norepinephrine (104). Understanding how key proteins involved in this signaling may lead to the development of novel treatments for stress-related disorders. Understanding how non-canonical signaling develops during chronic stress might reduce a wide range of stress-induced inflammatory processes.

The ultimate goal of understanding how inflammatory processes contribute to mental health is to develop novel treatments for stress-related mood disorders. This presents a considerable challenge as different individuals may have different pathologies driving inflammatory processes. For example, norepinephrine may be driving inflammation in some, whereas substance P may contribute to inflammation in others. A history of immune-related diseases may also complicate pathology. This challenge could potentially be overcome by using RNA sequencing to screen for changes in inflammatory transcripts. Transcriptional data could allow researchers to infer the signaling pathways that are driving inflammatory processes in stressed individuals. RNA sequencing technology is becoming more readily available and less expensive. Further, streamlined analysis pipelines are making it faster and easier to obtain meaningful results. This could help provide more personalized therapeutic strategies. Of course, this also has its own limitations as changes in transcription do not necessarily mean that changes in protein expression or function would be observed. Protein screens using multiplex enzyme-linked immunosorbent assays (ELISAs) might prove to be more useful, although they would not be able to screen as many targets as RNA sequencing would. Perhaps RNA sequencing of larger sample sizes scales could inform the development of more targeted and accurate multiplex ELISAs. Targeting differentially expressed proteins presents many of the same challenges as other brain disorders since the BBB limits the access of drugs to the brain. Targeting inflammatory cytokines in the periphery is more feasible, but their effects on mental health are more difficult to predict since not all of them directly contribute to the pathology of stress-related disorders.

A comprehensive understanding of the mechanisms underlying stress-induced infalmamtory processes may lay the groundwork for more targeted therapeutic strategies in treating stress-related disorders. For example, identifying intracellular signaling pathways by which noreinephrine increases inflammation might allow for treatments that are more specific to stress-induced inflammation without affecting noradrenergic neurotransmission or aspects of the innate immune response. Understanding mechanisms contributing to stress-induced inflammation may also inform therapeutic strategies for disorders of the immune system. For example, understanding glucocorticoid resistance and the psychiatric history of a patient might inform treatment strategies or dosage because individuals diagnosed with depression are less responsive to glucocorticoids (156). Adrenergic receptor agonists might be avoided and/or inflammatory markers might be more closely monitored when treating patients with a history of stress-related mental illness. As gene therapy becomes safer and more efficacious, alternatively expressed transcripts might be manipulated, especially for genes that cannot be specifically manipulated pharmacologically. This approach could be made more specific by targeting transcripts within specific cell types following screens for differentially expressed transcripts, allowing for more individualized treatments with minimal off-target effects. Further, chemogenetic and/or optogenetic methods could 1 day be use to manipulate noradrenergic neurotransmission in specific neuronal populations. An important consideration is that the use of adenoviruses, which have been commonly used vectors in gene therapy, can activate the innate immune response (183). Of course, this could negate manipulitons of gene expression that are intended to reduce inflammation. The use of lipid nanoparticles as vectors is promising because they are not always sufficient to induce an innate immune response, athough they may exacerbate the immune response to certain pathogenic stimuli (184). Understanding the crosstalk between nervous and immune systems could improve the specificity and efficacy of treatmens for a wide range of disorders related to stress and/or the immune system.

## Conclusion

This review examines the mechanisms by which stress affects inflammatory processes and how those inflammatory processes can alter behavior. Stress activates the HPA axis and sympathetic nervous system, which in turn activate anti-inflammatory GRs and pro-inflammatory NFkB, respectively. Stress-related mood disorders are characterized by glucocorticoid resistance and/or excess noradrenergic signaling, causing an imbalance that leads to increases in inflammatory processes. Preclinical work from our labs and others, as well as some human studies, has demonstrated that vulnerability to chronic stress is associated with inflammatory processes in the brain and periphery. These inflammatory processes contribute to anxiety- and depression-like behavior in rodents and depression severity scores in humans. Here, we review neuro-immune interactions and how they relate to pain circuitry as nociceptive signaling has an emotional component, which we believe should be thoughtfully considered in models of inflammatory effects on mental health. Additionally, a more comprehensive understanding of the specific cell types, circuits, and intracellular signaling mechanisms responsible for the inflammatory effects caused by norepinephrine is needed. In sum, this review aims to summarize our current understanding of how the immune and nervous systems respond to stress, communicate with one another, and regulate behavior. Understanding the pathology of stress-related mood disorders could lead to more personalized, efficacious treatments.

## Author contributions

RT, UK, LG, FH, NF, and BC performed literature searches and wrote the text. NF provided oversight on sections regarding pain. BC provided general oversight for this review. All authors contributed to the article and approved the submitted version.

## Funding

This work was funded in part by a Brain and Behavior Research Foundation (fka NARSAD) Young Investigator Award #29185, which was awarded to BC.

## Conflict of interest

The authors declare that the research was conducted in the absence of any commercial or financial relationships that could be construed as a potential conflict of interest.

## Publisher’s note

All claims expressed in this article are solely those of the authors and do not necessarily represent those of their affiliated organizations, or those of the publisher, the editors and the reviewers. Any product that may be evaluated in this article, or claim that may be made by its manufacturer, is not guaranteed or endorsed by the publisher.
